# The Elbert range of magnetostrophic convection. I. Linear theory

**DOI:** 10.1098/rspa.2022.0313

**Published:** 2022-08

**Authors:** Susanne Horn, Jonathan M. Aurnou

**Affiliations:** ^1^ Centre for Fluid and Complex Systems, Coventry University, Coventry CV1 5FB, UK; ^2^ Department of Earth, Planetary, and Space Sciences, University of California, Los Angeles, CA 90095, USA

**Keywords:** rotating convection, magnetoconvection, linear stability, dynamo

## Abstract

In magnetostrophic rotating magnetoconvection, a fluid layer heated from below and cooled from above is equidominantly influenced by the Lorentz and the Coriolis forces. Strong rotation and magnetism each act separately to suppress thermal convective instability. However, when they act in concert and are near in strength, convective onset occurs at less extreme Rayleigh numbers (Ra, thermal forcing) in the form of a stationary, large-scale, inertia-less, inviscid magnetostrophic mode. Estimates suggest that planetary interiors are in magnetostrophic balance, fostering the idea that magnetostrophic flow optimizes dynamo generation. However, it is unclear if such a mono-modal theory is realistic in turbulent geophysical settings. Donna Elbert first discovered that there is a range of Ekman (Ek, rotation) and Chandrasekhar (Ch, magnetism) numbers, in which stationary large-scale magnetostrophic and small-scale geostrophic modes coexist. We extend her work by differentiating five regimes of linear stationary rotating magnetoconvection and by deriving asymptotic solutions for the critical wavenumbers and Rayleigh numbers. Coexistence is permitted if $Ek<16/{\left(27\pi \right)}^{2}$ and $Ch\geq 27\pi^{2}$. The most geophysically relevant regime, *the Elbert range*, is bounded by the Elsasser numbers $\frac{4}{3}{\left(4^{4}\pi^{2}\;Ek\right)}^{1/3}\leq \Lambda \leq \frac{1}{2}{\left(3^{4}\pi^{2}Ek\right)}^{-1/3}$. Laboratory and Earth’s core predictions both exhibit stationary, oscillatory, and wall-attached multi-modality within the Elbert range.

## 1. Introduction

**Figure 1.**
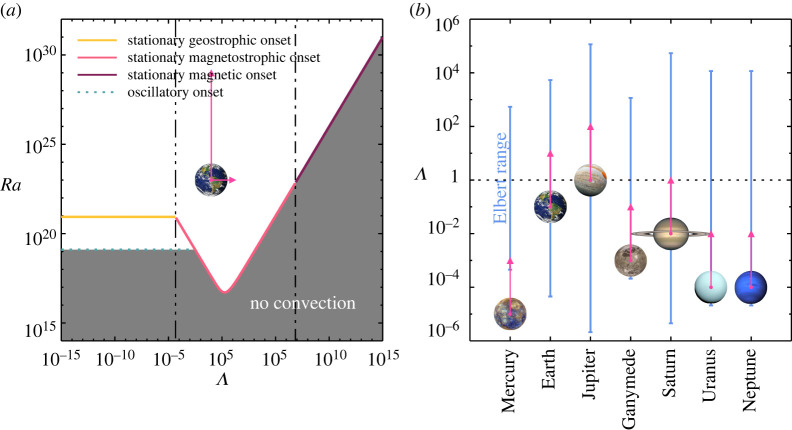
(*a*) Classical mono-modal linear stability prediction picture for Earth’s Ekman number of $Ek_{⊕}$ [9,12]. The solid lines mark the critical Rayleigh number Ra for the stationary onset of convection, corresponding to the geostrophic (yellow), magnetostrophic (magenta) or magnetic (merlot) solution branches. The dotted light blue line marks the oscillatory onset. The triple-dot-dashed vertical line demarcates $\Lambda_{\mathrm{sw}}$, the Elsasser number for mode switching between the small-scale geostrophic to the large-scale magnetostophic onset mode. The dot-dashed vertical line demarcates $\Lambda_{m}$, the Elsasser number at which the magnetostrophic onset mode is replaced by the magnetic one. The pink arrows indicate estimates for Earth’s Elsasser and Rayleigh number range, with the circle approximating the lower bounding value. The lower bound for Λ neglects contributions from the toroidal and unresolved poloidal components of the magnetic field, and thus, a more realistic estimate is about one order of magnitude higher [1,13,14]. (*b*) Estimates of the Elbert coexistence range indicated by the cornflower blue vertical lines for several celestial bodies, similar to (*a*), the pink symbols give estimates for Λ and circles indicate the lower bound [13,14]. (Online version in colour.)

Earth’s magnetic field is generated through convective motions in its molten metal outer core. These motions are affected by the Coriolis forces due to planetary rotation and by the Lorentz forces due to the dynamo-generated magnetic fields. A long held tenet of dynamo theory is that planetary magnetic field generation is optimized when the dynamics are in the magnetostrophic state in which Coriolis and Lorentz forces are approximately in balance [1–8]. This ‘magnetostrophic dynamo hypothesis’ is born out of the classical linear stability analysis [9–11], predicting that steady convection onsets most easily for magnetostrophy (figure 1*a*) and then occurs in the form of a large-scale bulk mode. It is supposed that dynamo action will be attracted to the magnetostrophic state where the most efficient dynamo generating flows are assumed to naturally exist. Furthermore, estimates for Earth suggest that the outer core is in the magnetostrophic state, since the intensity of the geomagnetic field appears to support this hypothesis [13,15]. The fortuitousness of the Earth lying in this apparent soft spot has made the stationary magnetostrophic mode the primary focus of a great deal of dynamo theory [1,4,8,11].

Magnetostrophic modes are also appealing because they do not depend on the fluid’s viscosity, suggesting that viscosity plays no role in this range [12]. Overly large viscous forces are considered the major shortcoming of present-day numerical simulations [7,14]. However, if the inviscid magnetostrophic hypothesis holds, then current-day simulation results may be meaningful nonetheless (cf. [8,16,17]).

A mono-modal magnetostrophic theory, however, is not likely to be geophysically realistic [18]. In rotating liquid metal convection without magnetic fields, even moderately supercritical flows are always strongly multi-modal. Oscillatory bulk modes, boundary-attached modes and stationary modes all exist simultaneously and interact nonlinearly [19–23]. Similarly, magnetoconvection without rotation is multi-modal with a mix of stationary bulk and boundary-attached modes [24–28]. Thus, unsurprisingly, multi-modality is also a characteristic feature of rotating magnetoconvection with a mix of stationary and oscillatory bulk modes, as well as drifting wall-attached modes [9,22,29,30].

The distinguishing and, at first glance, surprising feature of rotating magnetoconvection is that linear stability analysis predicts a more complex onset behaviour even for the stationary modes. In simple, rotating and magnetic Rayleigh–Bénard convection, exactly one mode describes the stationary marginal stability. By contrast, in rotating magnetoconvection two different types of stationary modes with length scales that are magnitudes apart can coexist for certain combinations of the rotation rate and the magnetic field strength [9].

**Figure 2.**
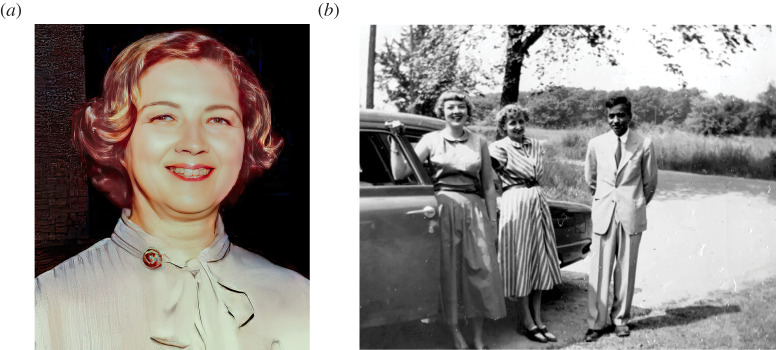
(*a*) Donna DeEtte Elbert (27 January 1928–15 January 2019). (*b*) Donna Elbert, Lillian Neff and Subrahmanyan Chandrasekhar, 1950 (left to right). Images courtesy of Dianne Hofner Saphiere, Susan Elbert Steele, Joanne Elbert Kantner. (Online version in colour.)

This magnetostrophic coexistence range was discovered by *Donna DeEtte Elbert* (figure 2). Elbert was Nobel laureate Subrahmanyan Chandrasekhar’s technical assistant and, at that time, was considered a ‘computer’. Nowadays, however, she would be recognized as a numerical and computational researcher in her own right. Elbert was the first to note these essential and most anomalous behaviours of the rotating magnetoconvection system that underlies magnetic field generation in geo- and astrophysical objects. This discovery is acknowledged as being hers in a footnote of Chandrasekhar’s seminal book *Hydrodynamic and Hydromagnetic Stability* [9, Chapter V.4, p. 219]:

*The fact that the curve R(a) defined by equations (59) and (60) has two minima for certain ranges of the parameters $Q_{1}$ and $T_{1}$ was first observed by Donna Elbert.*

In bringing these findings to Chandrasekhar, he was then able to lay the published ground work that is the theoretical foundation of the magnetostrophic dynamo hypothesis [1,10,12,31]. Dynamo researchers have been chasing and seeking to prove or disprove these ideas ever since [2–6,8,14,16,17,32–52]. Understanding the magnetostrophic coexistence range is essential for our view of planetary core convection as the magnetostrophic dynamo hypothesis relies on the nonlinear magnetohydrodynamics (MHD) in this range. We see in figure 1*b* that planetary dynamos in our solar system all likely exist in the coexistence range. Furthermore, the vast majority of numerical models of planetary dynamo action reside there as well.

### (a) Donna D. Elbert’s contributions to rotating magnetoconvective theory

Before delving into the details of rotating magnetoconvection, we will discuss the career and scientific contributions of Donna DeEtte Elbert of Williams Bay, Wisconsin (27 January 1928–15 January 2019), focusing on her 30-year collaboration with Chandrasekhar. In honour of her contributions, we will name the most geophysically relevant rotating magnetoconvective regime the *Elbert range*. The Elbert range is shown in figure 1*b*, together with quasi-static estimates of the Elsasser number for various celestial bodies.

Donna Elbert began working with Chandrasekhar at Yerkes Observatory in the autumn of 1948 as a ‘computer’, as people using electric-powered calculators were called at the time. She was 20 years of age and intended to work with Chandrasekhar only long enough to save up sufficient funds to attend design school. Elbert, however, remained Chandrasekhar’s research assistant, working at both Yerkes and the University of Chicago until 1979; their collaboration stayed fruitful over the years [53]. She started with numerical work on Heisenberg’s turbulence theory [54]. At the end of Chandrasekhar’s single author paper on this topic, he expresses his ‘indebtedness to Miss Donna Elbert for valuable assistance with the various numerical integrations…’, similar to acknowledgement statements he placed at the end of many of his papers over the next three decades. She worked with him on the polarization of the sunlit sky at about the same time, ultimately resulting in a co-authored journal article published in *Nature* and later on in the *Transactions of the American Philosophical Society* [53,55,56].

Elbert mastered numerical methods and was as tenacious and hard-working as Chandrasekhar himself [57]. Noting this, he encouraged her to take a series of advanced mathematics and calculus courses at the University of Wisconsin-Madison [58]. However, she never earned a formal degree in applied mathematics, but instead graduated years later, in 1974, from the School of the Art Institute of Chicago with a Bachelor of Fine Arts.

Especially in the years from 1948 to 1960, when Chandrasekhar worked on turbulence, MHD, rotating flows and convection, Elbert was indispensable and actively involved in this research [53]. Elbert co-authored 16 papers with Chandrasekhar, in which she carried out most of the numerical computations. Numerous times she developed solutions more elegant than Chandrasekhar’s original ones, e.g. finding a better solution ansatz for the roots of the dispersion relationship in rotating convection [9, Chapter III.2, p. 101]. According to Chandrasekhar’s autobiography, Elbert repeatedly revised his calculations ‘with unbounded patience with [his] errors’ [53]. Chandrasekhar also acknowledged that papers without her ‘numerical work [⋯] might not have been written’ [59] and that ‘her patience in carrying out the long (and often tiresome) calculations […] were necessary to obtain the concrete results’ [60]. In addition to her co-authored works with Chandrasekhar, Elbert also produced a single-authored publication discussing ‘Bessel and Related Functions which Occur in Hydromagnetics’ in the *Astrophysical Journal* [61], an impressive feat for a female automath in the 1950s.

Most pertinent in the present context, however, is her contribution to Chandrasekhar’s monograph *Hydrodynamic and Hydromagnetic Stability* [9], which is essential reading for those who study convection and instability. As stated in the preface [9, p. v]:

*I should, however, like to mention here the extent of my obligation to Miss Donna D. Elbert: in a real sense this book is the outcome of our joint efforts over the years and without her part there would have been no substance.*

Indeed, ‘Elbert carried out the relevant numerical calculations for most of the problems treated in this book; she is responsible for the numerical information included in all of the tables with the exception of Tables I-VI, X, XXII-XXX, XXXVI-XXXIX, XLVII, XLIX, LXIV-LXVII and LXX.’ That statement implies that Elbert carried out the calculations for 47 of the 70 tables in this 654 page treatise. If Chandrasekhar’s treatise [9] were being published now, Elbert would almost certainly have been a co-author (e.g. [62]).

During one of Chandrasekhar’s absences, on her own initiative, Elbert worked out the marginal stability curves for stationary rotating magnetoconvection [53]. These results are presented in Chapter V.4 *The thermal instability of a layer of fluid heated from below: The effect of rotation and magnetic field* in [9]. Elbert found that the marginal stability curves can contain two local minima, leading to a discontinuity at a critical magnetic field strength, and consequently to an abrupt change in the length scales of the convective onset modes. She also numerically determined that there exists a range of parameters over which small-scale geostrophic and large-scale magnetostrophic convective modes can coexist.

Donna Elbert’s findings will be at the core of the present paper. We extend her work by deriving asymptotically exact solutions for the wavenumbers and critical Rayleigh numbers in all the possible regimes of stationary rotating magnetoconvection. We also derive novel analytical expressions for the asymptotic bounds of Elbert’s coexistence range of stationary rotating magnetoconvective modes. We use these linear stability analysis results to give predictions for laboratory experiments and for Earth’s core.

## 2. Governing linear equations of rotating magnetoconvection

We will present the results of a linear stability analysis carried out in the spirit of Chandrasekhar [9] and Eltayeb [12]. Asymptotically exact solutions are obtained using Laurent series expansions. No attempt shall be made here to trace exact bifurcation scenarios. Rather our aim is to give predictions for when to expect transitions in supercritical, turbulent settings. This is motivated by the succesful application of this approach to rotating convection in liquid metals [19–21], where we showed that these predictions give sufficiently accurate estimates of when changes occur in flow morphology, including in the spatio-temporal scales, and in the heat and momentum transport. Furthermore, we found that the signatures of the underlying instability mechanisms remain present up to relatively high supercriticalities. This suggests that our results are relevant in informing our understanding of planetary core convection [1,14].

We analyse the linear stability of a fluid in a (semi-)infinite plane layer with free-slip, isothermal and electrically insulating boundary conditions. Asymptotically, i.e. with sufficiently rapid rotation and strong magnetic field, the results do not depend on the mechanical, thermal or magnetic boundary conditions at leading order. But deviations can be expected for less extreme parameters due, for example, to viscous and Ekman–Hartmann boundary layers [12,63–71]. In part II of this series, we will discuss corresponding nonlinear numerical simulations and also compare them to experimental findings.

We consider a fluid with kinematic viscosity ν, thermal diffusivity κ, magnetic diffusivity η, density ρ and electrical conductivity σ. The fluid layer has thickness H, is heated from below and is cooled above, resulting in a temperature difference Δ. The layer is also subject to angular velocity $\Omega =\Omega {\hat{e}}_{z}$ and magnetic field $B=B{\hat{e}}_{z}$ vectors that are both aligned in the vertical ${\hat{e}}_{z}$-direction.

We seek to determine the marginal state at which the conductive state becomes unstable to convective motions, i.e. the onset of convection. The governing non-dimensional linear equations of rotating magnetoconvection for the velocity field $u=(u_{x},u_{y},u_{z})$, the deviation of the temperature from the conductive solution θ, pressure p and induced magnetic field b read [9,12]

2.1 $$\begin{array}{rl} & \;(\partial_{t}-\nabla^{2})u=-\nabla p-Ek^{-1}({\hat{e}}_{z}\times u)+Ch({\hat{e}}_{z}\cdot \nabla )b+Ra\;\theta {\hat{e}}_{z}, \end{array}$$

2.2 $$\begin{array}{rl} & \;-\nabla^{2}b=({\hat{e}}_{z}\cdot \nabla )u, \end{array}$$

2.3 $$\begin{array}{rl} & \;(Pr\;\partial_{t}-\nabla^{2})\theta =u_{z}, \end{array}$$

2.4 $$\begin{array}{rl} \mathrm{and}\; & \nabla \cdot u=\nabla \cdot b=0, \end{array}$$

with the reference scales H, Δ, B and $H^{2}/\nu$ for time. Here, we have employed the Oberbeck–Boussinesq approximation [72–75] including rotation in the co-rotating frame of reference, neglecting the centrifugal buoyancy force [76,77], and magnetic fields in the quasi-static MHD approximation [78,79]. The main idea behind the quasi-static MHD approximation is that the induced magnetic field b is negligible compared to the imposed external field B, and further fluctuations are assumed to adapt instantaneously to the slowly varying velocity field, i.e. $\partial_{t}b\approx 0$. This implies a small magnetic Reynolds number Rm=|u|H/η≪1 and a negligible magnetic Prandtl number Pm=ν/η≪1. The low-Rm approximation does not allow for self-generated magnetic fields and consequently dynamo action, and it does also not permit magnetic overstability [42]. However, the approximation is applicable for small-scale planetary core flows [80] and also liquid metal experiments [22,81]. The finite-Pm case for the onset of oscillatory convection is discussed in more detail by Chandrasekhar [9] and Julien *et al.* [42]. The stationary onset predictions are not affected; they yield the same result under the quasi-static approximation and in the full MHD system.

The non-dimensional control parameters appearing in the governing equations (2.1)–(2.4) are the Rayleigh, Prandtl, Ekman and Chandrasekhar numbers

2.5 $$Ra=\frac{\alpha g\Delta H^{3}}{\kappa \nu },\;Pr=\frac{\nu }{\kappa },\;Ek=\frac{\nu }{2\Omega H^{2}}\;\mathrm{and}\;Ch=\frac{\sigma B^{2}H^{2}}{\rho \nu }.$$

In the linear analysis, the balance of Lorentz and Coriolis forces is expressed as the quasi-static Elsasser number [3]

2.6 $$\Lambda =\frac{\sigma B^{2}}{2\rho \Omega }=Ch\;Ek.$$

Alternative control parameters found in the literature include the Rossby, Taylor, Hartmann and Stuart numbers:

2.7 $$Ro=\sqrt{\frac{Ra\;Ek^{2}}{Pr}},\;Ta=\frac{1}{Ek^{2}},\;Ha=\sqrt{Ch}\;\mathrm{and}\;N=\sqrt{\frac{Ch^{2}\;Pr}{Ra}}.$$

The standard approach is to determine convective onset by seeking solutions in the form of normal modes $F(x,y,z,t)=F(z)\exp(\text{i}(a_{x}x+a_{y}y+\sigma t))$ where F represents any of the variables θ, u, p and b. The non-dimensional horizontal wavenumber is given by $a=\sqrt{a_{x}^{2}+a_{y}^{2}}$ and σ is the non-dimensional frequency. Here, for example, $a_{x}\equiv n\pi$ where n is the non-negative integer mode number in the ${\hat{e}}_{x}$-direction. Note also that we will convert σ to the more convenient non-dimensional frequency ω, which is normalized by the rotation rate, i.e. ω=2σ/Ek. As shown in detail by Chandrasekhar [9] as well as Eltayeb [12], the solution to the linear stability problem amounts to finding the solution of $u_{z}$, all other variables are expressed in terms of it. This gives $u_{z}=\cos(n\pi z)$ for our set of boundary conditions. The most easily excited mode always has a vertical modenumber of n=1. Higher n modes are excited at higher Ra, but this is beyond the scope of the present study. Throughout the paper, we will start with the marginal stability relation, obtained by substituting in the normal modes into equations (2.1)–(2.4) (e.g. [82]). The critical Rayleigh number $Ra_{\text{crit}}$ is then determined as the minimum of this curve, along with the corresponding wavenumber $a_{\text{crit}}$ and, if applicable, $\omega_{\text{crit}}$. The set $\left(Ra_{\text{crit}},a_{\text{crit}},\omega_{\text{crit}}\right)$ then defines the most easily excited linear convective mode [9,12].

To further simplify the analysis, we introduce auxilliary variables similar to Chandrasekhar [9]:

2.8 $$Ek_{1}=Ek\;\pi^{2},\;Ch_{1}=\frac{Ch}{\pi^{2}},\;\Lambda =Ch\;Ek=Ch_{1}Ek_{1}\;\mathrm{and}\;k=1+\frac{a^{2}}{\pi^{2}}.$$

Traditionally, the modified wavenumber k is chosen to be $a^{2}/\pi^{2}$, thus, all the polynomial expressions given here are novel, albeit yielding the same solutions as previous formulations.

The expected instability mechanisms occur in the form of stationary, oscillatory and wall-attached convection modes. We refer to this variety of modes as multi-modality and only discuss the respective onset mode, notwithstanding that at higher supercriticalities several modes of each type become unstable and are excited. We will focus on typical liquid metals, that is Pr<1, but Pm≪Pr, in agreement with the quasi-static approximation. Thus, we allow for oscillatory convection originating from the Coriolis but not from the Lorentz force.

An interactive python Jupyter Notebook containing the full solutions $\left(Ra_{\text{crit}},a_{\text{crit}},\omega_{\text{crit}}\right)$ for all the convective onset modes is provided as electronic supplementary material.

## 3. Linear stability of stationary rotating magnetoconvection

The onset of stationary convection is a fairly straightforward problem for Rayleigh–Bénard convection, rotating convection and magnetoconvection. Analysis of the linear equations provides exactly one global minimum and, hence, only one critical Rayleigh number for marginal stability.

By contrast, in rotating magnetoconvection, the situation is more complicated. On their own, both rotation and magnetic fields act to suppress convection and several of the results can be carried over from one system to the other (e.g. [83]). The most significant differences are that the magnetic field provides an additional source of dissipation because it effectively acts as an additional, anisotropic viscosity [84,85], but unlike rotation it does not induce vorticity and does not break horizontal symmetry [86]. As a consequence, magnetic fields and rotation acting together have conflicting tendencies and, nonintuitively, can facilitate convection when the Lorentz and Coriolis forces are approximately in balance [10].

The critical Rayleigh number $Ra_{s}$ for the stationary onset is determined by minimization of Chandrasekhar [9]

3.1 $$Ra=\frac{a^{2}+\pi^{2}}{a^{2}}\left({\left(a^{2}+\pi^{2}\right)}^{2}+\pi^{2}Ch+\frac{\pi^{2}(a^{2}+\pi^{2})}{Ek^{2}({\left(a^{2}+\pi^{2}\right)}^{2}+\pi^{2}Ch)}\right).$$

**Figure 3.**
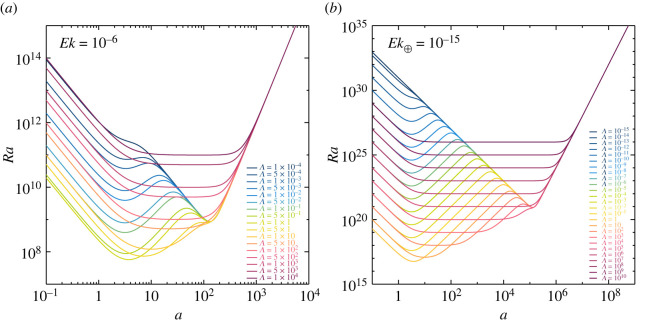
Stationary marginal Rayleigh number Ra as a function of wavenumber a according to equation (3.1). There are either one minimum, or two local minima and a local maximum, depending on the value of the quasi-static Elsasser number Λ for (*a*) a laboratory-like Ekman number, $Ek=10^{-6}$, (*b*) an Earth-like $Ek_{⊕}=10^{-15}$. (Online version in colour.)

These curves are shown in figure 3*a* for a typical laboratory-numerical value of $Ek=10^{-6}$ and in figure 3*b* for an Earth-like value of $Ek=10^{-15}$. For small or large Elsasser numbers, the marginal Ra curves contain only one minimum. For Λ≪1, rotational forces dominate and convection onsets in the form of high wavenumber geostrophic modes. For Λ≫1, magnetic forces dominate and convection onsets in the form of relatively high wavenumber magnetic modes. In the intermediate Elsasser number range, Λ∼1, the marginal curves feature two local minima, separated by a local maximum. As noted first by Donna Elbert [9], the two local minima imply the coexistence of two distinct linearly unstable modes with $a_{c}$ values that can be many magnitudes apart. The leftward minima, located near a=π in each panel, corresponds to the large-scale magnetostrophic mode while the rightward minima correspond to the higher wavenumber, smaller-scale geostrophic mode. Following this, Eltayeb [12] proposed that three main regimes exist, one where rotation dominates, one where the magnetic field dominates and another one where both are equidominant.

Here, instead, we will show that a full description of linear, stationary rotating magnetoconvection requires five separate regimes, two of which feature coexisting dual-mode stationary solutions. The onset of stationary rotating magnetoconvection is determined through $\partial_{k}Ra=0$. This leads to a septic polynomial equation in (k, $Ek_{1}$, $Ch_{1}$),

3.2 $$\begin{array}{rl} & k^{7}-\frac{3}{2}k^{6}+2Ch_{1}k^{5}-\frac{7Ch_{1}Ek_{1}^{2}+1}{2Ek_{1}^{2}}k^{4}+Ch_{1}^{2}k^{3}\\ & \;\;+\frac{Ch_{1}-5Ch_{1}^{2}Ek_{1}^{2}}{2Ek_{1}^{2}}k^{2}-\frac{Ch_{1}}{Ek_{1}^{2}}k-\frac{Ch_{1}^{3}}{2}=0, \end{array}$$

which can also be expressed in terms of (k, $Ek_{1}$, Λ) as

3.3 $$\begin{array}{rl} & k^{7}-\frac{3}{2}k^{6}+\frac{2\Lambda }{Ek_{1}}k^{5}-\frac{7Ek_{1}\Lambda +1}{2Ek_{1}^{2}}k^{4}+\frac{\Lambda^{2}}{Ek_{1}^{2}}k^{3}\\ & \;\;+\frac{\Lambda -5Ek_{1}\Lambda^{2}}{2Ek_{1}^{3}}k^{2}-\frac{\Lambda }{Ek_{1}^{3}}k-\frac{\Lambda^{3}}{2Ek_{1}^{3}}=0, \end{array}$$

or in terms of (k, $Ch_{1}$, Λ) as

3.4 $$\begin{array}{rl} & k^{7}-\frac{3}{2}k^{6}+2Ch_{1}k^{5}-\frac{Ch_{1}(Ch_{1}+7\Lambda^{2})}{2\Lambda^{2}}k^{4}+Ch_{1}^{2}k^{3}\\ & \;\;+\frac{Ch1^{2}(Ch1-5\Lambda^{2})}{2\Lambda^{2}}k^{2}-\frac{Ch_{1}^{3}}{\Lambda^{2}}k-\frac{Ch_{1}^{3}}{2}=0. \end{array}$$

The three formulations above allow for the analysis of different asymptotic limits, which demarcate the five separate stationary regimes in the following subsections.

**Figure 4.**
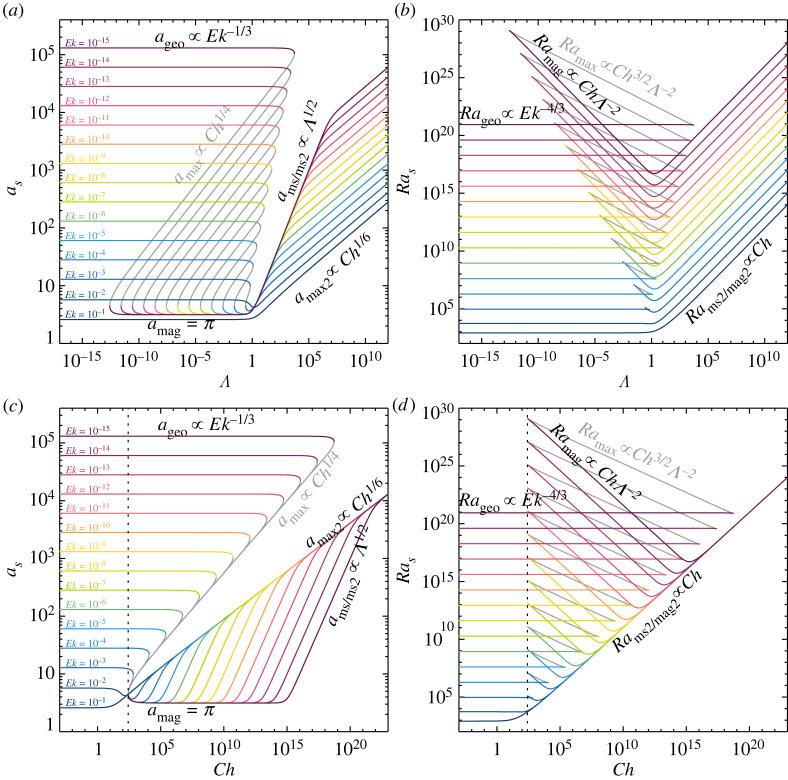
(*a*) Critical wavenumber $a_{s}$ and (*b*) corresponding Rayleigh number $Ra_{s}$ as a function of the Elsasser number Λ for $10^{-15}\leq Ek\leq 10^{-1}$ for stationary rotating magnetoconvection. (*c*) Critical wavenumber $a_{s}$ and (*d*) corresponding Rayleigh number $Ra_{s}$, similar to (*a*,*b*), but here as a function of the Chandrasekhar number Ch. The grey lines correspond to the local maximum of the marginal Ra curve (3.1). The vertical dashed black lines in (*c*,*d*) mark the minimum Chandrasekhar number for modal coexistence, $Ch_{\text{lc}}=27\pi^{2}$ (3.11). The leading order asymptotic scalings are indicated in the figure and are summarized in table 1. (Online version in colour.)

**Figure 5.**
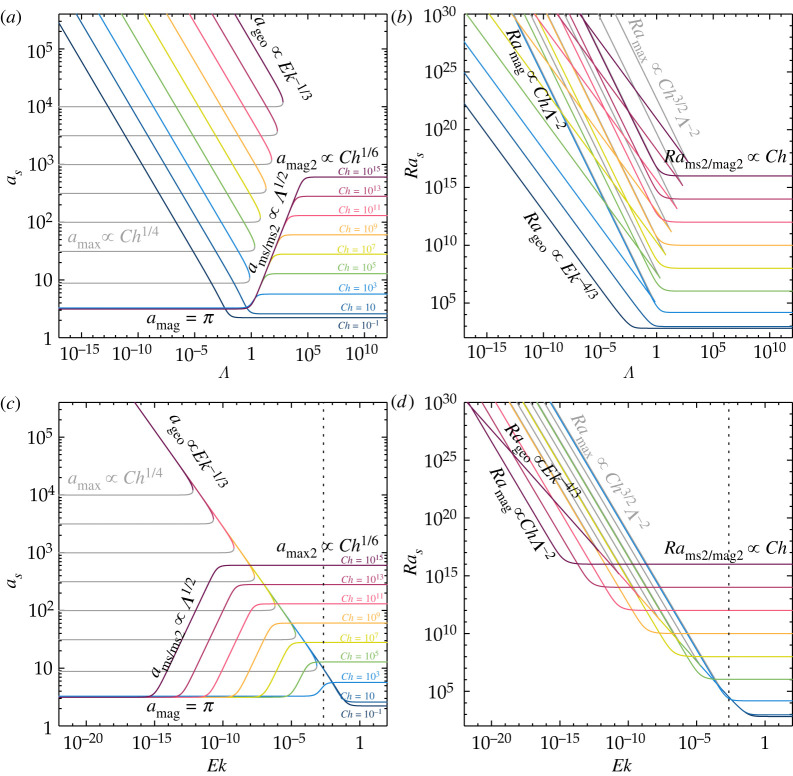
(*a*) Critical wavenumber $a_{s}$ and (*b*) corresponding Rayleigh number $Ra_{s}$ as a function of the Elsasser number Λ for $10^{-1}\leq Ch\leq 10^{15}$. (*c*) Critical Wavenumber $a_{s}$ and (*d*) corresponding Rayleigh number $Ra_{s}$ as a function of the Ekman number Ek for $10^{-1}\leq Ch\leq 10^{15}$. The grey lines correspond to the local maximum of the marginal Ra curve (3.1). The vertical dashed black lines in (*c*,*d*) mark the maximum Ekman number for modal coexistence, $Ek^{*}={\left(2^{2}/(3^{3}\pi )\right)}^{2}$ (3.18). The leading order asymptotic scalings are indicated in the figure and are summarized in table 1. (Online version in colour.)

The polynomial representation, used throughout this study and in the accompanying Jupyter Notebook, has noteworthy advantages. Numerical root finding algorithms for polynomials are extremely robust, fast and accurate [87]. Hence, the polynomial representation is preferable over the classical way of finding the minimal value via a search over a range of different wavenumbers k. The full solution in terms of the critical wavenumbers $a_{s}$ and Rayleigh numbers $Ra_{s}$ is shown in figures 4 and 5. These solutions are obtained numerically and reveal fundamentally different behaviours in different parameter ranges.

**Table 1.** Leading order asymptotic behaviours of the critical wavenumbers and Rayleigh numbers in the five regimes of stationary rotating magnetoconvection elucidated herein, see figure 6*c*,*d*. Many of these relations can alternatively be expressed in terms of Ch via Λ=Ek Ch. The boundaries between the regimes are given by $\Lambda_{\mathrm{lc}}=27\pi^{2}Ek=266.5\;Ek$, $\Lambda_{\mathrm{sw}}=\frac{4}{3}{\left(4\pi^{2}Ek\right)}^{1/3}=4.540\;Ek^{1/3}$, $\Lambda_{\mathrm{uc}}=\frac{1}{2}{\left(3^{4}\pi^{2}Ek\right)}^{-1/3}=0.054Ek^{-1/3}$, $\Lambda_{m}=\frac{1}{2^{1/2}\pi }Ek^{-1/2}=0.225Ek^{-1/2}$.

regime	Elsasser range	$a_{\mathrm{crit}}$	$Ra_{\mathrm{crit}}$
G	$\Lambda <\Lambda_{\mathrm{lc}}$	$a_{\mathrm{geo}}=\frac{\pi^{1/3}}{2^{1/6}}\;Ek^{-1/3}$	$Ra_{\mathrm{geo}}=\frac{3\pi^{4/3}}{2^{2/3}}\;Ek^{-4/3}$
		$=1.305\;Ek^{-1/3}$	$=8.696\;Ek^{-4/3}$
${\mathrm{MG}}_{1}$	$\Lambda_{\mathrm{lc}}\leq \Lambda <\Lambda_{\mathrm{sw}}$	$a_{\mathrm{geo}}=\frac{\pi^{1/3}}{2^{1/6}}\;Ek^{-1/3}$	Rageo=3π4/322/3 Ek−4/3aasass
		$a_{\mathrm{mag}}=\pi$	$Ra_{\mathrm{mag}}=4\pi^{2}\frac{Ch}{\Lambda^{2}}$
		$a_{\max}=\pi^{1/2}\;Ch^{1/4}$	Ramax=π2Ch3/2Λ2aasass
${\mathrm{MG}}_{2}$	$\Lambda_{\mathrm{sw}}\leq \Lambda <\Lambda_{\mathrm{uc}}$	$a_{\mathrm{geo}}=\frac{\pi^{1/3}}{2^{1/6}}\;Ek^{-1/3}$	Rageo=3π4/3aasass22/3 Ek−4/3
(Elbert range)		$a_{\mathrm{ms}}=\pi {\left(1+\Lambda^{2}\right)}^{1/4}$	$Ra_{\mathrm{ms}}=\frac{\pi^{2}{\left(1+\sqrt{1+\Lambda^{2}}\right)}^{2}}{\Lambda Ek}$
		$a_{\max}=\pi^{1/2}\;Ch^{1/4}$	Ramax=π2Ch3/2aasassΛ2
${\mathrm{MG}}_{3}$	$\Lambda_{\mathrm{uc}}\leq \Lambda <\Lambda_{m}$	$a_{\mathrm{ms}2}=\pi \Lambda^{1/2}$	$Ra_{\mathrm{ms}2}=\pi^{2}Ch$
M	$\Lambda >\Lambda_{m}$	$a_{\mathrm{mag}2}=\frac{\pi^{2/3}}{2^{1/6}}Ch^{1/6}$	$Ra_{\mathrm{mag}2}=\pi^{2}Ch$
		$=1.911\;Ch^{1/6}$	

**Figure 6.**
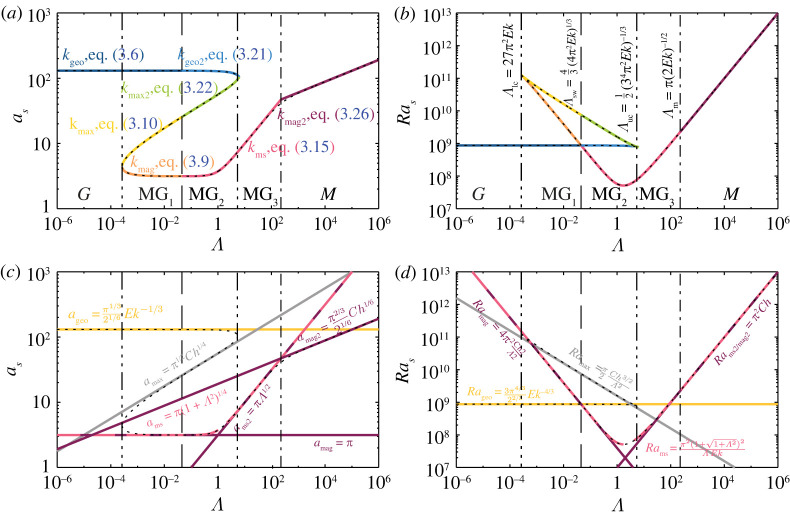
Solution of the stationary rotating magnetoconvection linear stability problem at $Ek=10^{-6}$. The dotted black curves show the solution of the full septic polynomial (3.2). (*a*,*c*) Critical wavenumbers $a_{s}$. (*b*,*d*) Critical Rayleigh numbers $Ra_{s}$. The different colours in (*a*,*b*) denote the various asymptotic solutions calculated using the reduced wavenumbers $k=1+{\left(a/\pi \right)}^{2}$ (i.e. $a=\pi \sqrt{k-1}$), as shown in (*a*) and discussed in §§3a–e. (*c*,*d*) Similar to (*a*,*b*), but showing the leading order terms of the asymptotic solutions (table 1); the yellow solid line gives the geostrophic, the merlot solid lines the magnetic, and the pink dashed line the magnetostrophic solutions in each panel. The vertical lines in each panel indicate the regime boundaries between the geostrophic range (G), the geostrophic coexistence range (${\mathrm{MG}}_{1}$), Elbert’s magnetostrophic coexistence range (${\mathrm{MG}}_{2}$), the magnetically dominated magnetostrophic range (${\mathrm{MG}}_{3}$) and the magnetic range (M). (Online version in colour.)

There is no general algebraic solution, according to the Abel–Ruffini theorem, for a polynomial of degree five or higher. This necessitates that we develop asymptotic solutions (figures 4–6) based on series expansions in the following analyses of stationary rotating magnetoconvection. These asymptotic expansions yield lower-order polynomials that describe the behaviour of the critical Rayleigh numbers and wavenumbers in our five ranges: the geostrophic (G) range, the geostrophic coexistence (${\mathrm{MG}}_{1}$) range, the magnetostrophic coexistence (${\mathrm{MG}}_{2}$) range, the magnetically dominated magnetostrophic (${\mathrm{MG}}_{3}$) range and the magnetic (M) range.

The magnetostrophic coexistence (${\text{MG}}_{2}$) range is the most relevant regime in geophysics and planetary physics, and, thus, we choose to christen it *the Elbert range* in honour of Donna Elbert’s seminal contributions to the field.

### (a) The geostrophic range (G)

The geostrophic range (G) is characterized by a negligible magnetic field. Formally it exists in the limit of Ch→0, or, alternatively, if Lorentz forces are weak compared to the Coriolis forces, i.e. Λ→0. Then a zeroth order series expansion of either the polynomial (3.2) or (3.3), respectively, yields the cubic polynomial,

3.5 $$k^{3}-\frac{3k^{2}}{2}-\frac{1}{2Ek_{1}^{2}}=0.$$

Polynomial (3.5) is identical to the one describing the onset of purely rotating convection [9,88] and has only one real valued root,

3.6 $$k_{\text{geo}}=\frac{1}{2}\left(\frac{{\left(\sqrt{Ek_{1}^{2}+1}+1\right)}^{2/3}}{Ek_{1}^{2/3}}+\frac{{\left(\sqrt{Ek_{1}^{2}+1}-1\right)}^{2/3}}{Ek_{1}^{2/3}}+1\right).$$

We can insert (3.6) into (3.1) to obtain the asymptotic critical wavenumber and Rayleigh number (figure 6*a*,*b*). In the limit of rapid rotation, $Ek_{1}\to 0$, the leading order term yields the familiar result

3.7 $$a_{\text{geo}}=\frac{\pi^{1/3}}{2^{1/6}}Ek^{-1/3}\;\mathrm{and}\;Ra_{\text{geo}}=\frac{3\pi^{4/3}}{2^{2/3}}Ek^{-4/3}$$

(figure 6*c*,*d*). Under many circumstances the leading order term gives a sufficiently accurate approximation [68,89,90] and it is this leading order term that is independent of different boundary conditions [65].

### (b) The geostrophic coexistence range (${\mathrm{MG}}_{1}$)

The onset of convection can also occur in the form of modes that are predominantly geostrophic but whose behaviour is also non-negligibly determined by magnetic effects. Two types of modes occur in this geostrophic coexistence range. One is the geostrophic mode determined above by $a_{\text{geo}}$ and $Ra_{\text{geo}}$ of equation (3.7), which is the onset mode in this range. In addition, there is a magnetic mode. In the limit $Ek_{1}\to 0$ or Λ→0, a zeroth order series expansion of the respective polynomials (3.2) or (3.4) yields the cubic polynomial

3.8 $$k^{3}-Ch_{1}k+2Ch_{1}=0.$$

This polynomial has real-valued solutions with k≥1 only for $Ch_{1}\geq 27$. There are two viable solutions for $Ch_{1}>27$. The first one corresponds to the magnetic branch,

3.9 $$k_{\text{mag}}=\frac{{\left(-3\right)}^{2/3}Ch_{1}-{\left(-3\right)}^{1/3}{(\sqrt{3(27-Ch_{1})}-9)}^{2/3}Ch_{1}^{2/3}}{3{(\sqrt{3(27-Ch_{1})}-9)}^{1/3}Ch_{1}^{1/3}},$$

because it only depends on $Ch_{1}$ (throughout this paper, we will use principal roots, e.g. ${\left(-3\right)}^{1/3}=(3^{1/3}+i3^{5/6})/2$). The other real-valued solution corresponds to the local maximum of the Rayleigh number,

3.10 $$k_{\text{max}}=\frac{3^{1/3}Ch_{1}+{(\sqrt{3(27-Ch_{1})}-9)}^{2/3}Ch_{1}^{2/3}}{3^{2/3}{(\sqrt{3(27-Ch_{1})}-9)}^{1/3}Ch_{1}^{1/3}}.$$

While not corresponding to a physical mode, $k_{\text{max}}$ gives insight into how the geostrophic and magnetic/magnetostrophic modes are connected (figure 6). For $Ch_{1}=27$, both solutions simplify to $k_{\mathrm{mag}/\max}=3$. The corresponding critical Rayleigh numbers and wavenumbers can be determined accordingly.

The lower boundary of the coexistence range (lc), i.e. the Chandrasekhar or Elsasser number, respectively, below which magnetic modes cease to exist, is

3.11 $$G⇆{\text{MG}}_{1}:\;Ch_{\text{lc}}=27\pi^{2}\Leftrightarrow \Lambda_{\text{lc}}=27\pi^{2}Ek.$$

Conversely, the lower bound of the coexistence range means that the geostrophic regime (G) can only exist in systems with $Ch<Ch_{\text{lc}}$. If $Ch\geq Ch_{\text{lc}}$, geostrophic modes do not exist mono-modally, but instead must be coexistent with magnetic and magnetostrophic modes.

Expressions (3.9) and (3.10) provide asymptotic relations in the limit of $Ch_{1}\to \infty$ with the following leading order terms

3.12 $$a_{\text{mag}}=\pi ,\;Ra_{\text{mag}}=4\pi^{2}\frac{Ch}{\Lambda^{2}};$$

and

3.13 $$a_{\text{max}}=\pi^{1/2}Ch^{1/4},\;Ra_{\text{max}}=\frac{\pi }{2}\frac{Ch^{3/2}}{\Lambda^{2}}.$$

Interestingly, the critical Rayleigh numbers of these magnetic branches, $Ra_{\text{mag}}$ and $Ra_{\text{max}}$, depend on Ch, Ek and Λ, even though $k_{\text{mag}}$ and $k_{\text{max}}$ depend only on Ch.

### (c) The Elbert magnetostrophic coexistence range (${\mathrm{MG}}_{2}$)

The key feature of ${\text{MG}}_{2}$, the Elbert range, is the existence of a large-scale, inertia-less, inviscid magnetostrophic mode, which has long been purported to solely dominate dynamo action in planetary core settings (e.g. [1]). Classically, the regime boundary between ${\text{MG}}_{1}$ and ${\text{MG}}_{2}$ is the onset mode switching point $\Lambda_{\text{sw}}$ (as defined below), corresponding to the well-known discontinuity in the critical Rayleigh number curve, $Ra_{s}(\Lambda )$. In ${\text{MG}}_{1}$, the geostrophic mode is the first to onset. In ${\text{MG}}_{2}$, the magnetostrophic mode is the most linearly unstable mode of stationary rotating magnetoconvection. This has led to the idea that the magnetostrophic mode replaces the geostrophic mode in ${\text{MG}}_{2}$ and is the only mode relevant to dynamo action in the Λ∼1 regime. However, as first elucidated by Donna Elbert, the geostrophic mode does not disappear but instead, at sufficiently high Ra, coexists with the magnetostrophic mode, likely leading to multi-modal magnetostrophic turbulence.

The mathematical description of the asymptotic behaviour in the Elbert range requires taking the double limit $Ch_{1}\to \infty$ and $Ek_{1}\to 0$, i.e. simultaneously assuming that the magnetic field is strong and the rotation is rapid. This is best achieved by keeping $\Lambda =Ch_{1}Ek_{1}$ a finite constant and then taking a single limit. Hence, the critical mode in the Elbert range is derived by taking either the limit of $Ek_{1}\to 0$ of the full polynomial (3.3) expressed in terms of $Ek_{1}$ and Λ or the limit of $Ch_{1}\to \infty$ of the full polynomial (3.4) expressed in terms of $Ch_{1}$ and Λ. In either case, we arrive at the simple quadratic polynomial,

3.14 $$k^{2}-2k-\Lambda^{2}=0.$$

The leads to the famous magnetostrophic solution [1,12]

3.15 $$k_{\text{ms}}=1+\sqrt{1+\Lambda^{2}},$$

resulting in the critical wavenumber and the critical Rayleigh number

3.16 $$a_{\text{ms}}=\pi {\left(1+\Lambda^{2}\right)}^{1/4},\;Ra_{\text{ms}}=\frac{\pi^{2}{\left(1+\sqrt{1+\Lambda^{2}}\right)}^{2}}{\Lambda \;Ek}=\frac{\pi^{2}{\left(1+\sqrt{1+\Lambda^{2}}\right)}^{2}Ch}{\Lambda^{2}}.$$

For Λ→0, the magnetostrophic solution (3.16) connects smoothly to the magnetic solution (3.12) in ${\text{MG}}_{1}$, such that $a_{\text{ms}}\to a_{\text{mag}}$ and $Ra_{\text{ms}}\to Ra_{\text{mag}}$, as can be seen in figure 6*c*,*d*.

The large-scale magnetostrophic mode $\left(a_{\text{ms}},Ra_{\text{ms}}\right)$ supersedes the small-scale geostrophic mode $\left(a_{\text{geo}},Ra_{\text{geo}}\right)$ as onset mode at the mode switching Elsasser number $\Lambda_{\text{sw}}$. It is determined by the condition that the transition from the geostrophic coexistence range ${\text{MG}}_{1}$ to Elbert’s magnetostrophic coexistence range ${\text{MG}}_{2}$ is smooth [1,12], i.e. where $Ra_{\text{geo}}=Ra_{\text{ms}}$. However, the solution of $Ra_{\text{geo}}=Ra_{\text{ms}}$ is difficult to attain analytically. Using the asymptotic expression $Ra_{\text{mag}}$ in place of $Ra_{\text{ms}}$ then yields the known mode switching result [1,12] that marks the border between ${\text{MG}}_{1}$ and ${\text{MG}}_{2}$,

3.17 $${\text{MG}}_{1}⇆{\text{MG}}_{2}:\;\Lambda_{\text{sw}}=\frac{4}{3}{\left(4\pi^{2}Ek\right)}^{1/3}=\frac{16\pi }{3^{3/2}}Ch^{-1/2}.$$

In addition, relation (3.17) allows us to determine the maximal Ekman number $Ek^{*}$ below which the coexistence range emerges,

3.18 $$\Lambda_{\text{lc}}=\Lambda_{\text{sw}}\Rightarrow Ek^{*}={\left(\frac{2^{2}}{3^{3}\pi }\right)}^{2}.$$

Prior to this work, $Ek^{*}=16/{\left(27\pi \right)}^{2}=2.223\times 10^{-3}$ had to be approximated numerically [1,9], without analytical justification.

The overall minimum of the critical Rayleigh number and the corresponding Elsasser and wavenumber [1] is found via $\partial_{\Lambda }Ra_{\text{ms}}=0$, viz.

3.19 $$\Lambda_{\text{min}}=3^{1/2},\;Ra_{\text{min}}=3^{3/2}\pi^{2}Ek^{-1}\;\mathrm{and}\;a_{\text{min}}=\sqrt{2}\pi .$$

In decimal form, the relations for the overall minimum correspond to $\Lambda_{\text{min}}=1.732$, $Ra_{\text{min}}=51.28\;Ek^{-1}$ and $a_{\text{min}}=4.443$.

The leading order approximation to the geostrophic and maximum modes are, respectively, given by $\left(a_{\text{geo}},Ra_{\text{geo}}\right)$, equation (3.7), and $\left(a_{\text{max}},Ra_{\text{max}}\right)$, equation (3.13), as shown in figure 6*c*,*d*. In contrast to the accurate corresponding solutions (3.9) and (3.10) that capture the connection between the magnetic branch and the maximum branch in ${\text{MG}}_{1}$, these leading order terms do not capture the connection point between the geostrophic branch and the maximum branch in ${\text{MG}}_{2}$ (figure 6*c*). Consequentially, we cannot use equations (3.7) and (3.13) to determine the upper boundary, $\Lambda_{\text{uc}}$, of Elbert’s magnetostrophic coexistence range, ${\text{MG}}_{2}$.

In order then to find $\Lambda_{\text{uc}}$, we divide the full septic polynomial (3.3) by the quadratic polynomial (3.14). This yields a quintic polynomial and gives the proper asymptotic solution for the geostrophic and maximum branches in ${\text{MG}}_{2}$,

3.20 $$\begin{array}{rl} & k^{5}+\frac{1}{2}k^{4}+(1+\frac{2\Lambda }{Ek_{1}}+\Lambda^{2})k^{3}+(2-\frac{1}{2Ek_{1}^{2}}+\frac{\Lambda }{2Ek_{1}}+\frac{5\Lambda^{2}}{2})k^{2}\\ & \;\;+(4-\frac{1}{Ek_{1}^{2}}+\frac{\Lambda }{Ek_{1}}+6\Lambda^{2}+\frac{\Lambda^{2}}{Ek_{1}^{2}}+\frac{2\Lambda^{3}}{Ek_{1}}+\Lambda^{4})k\\ & \;\;+8-\frac{2}{Ek_{1}^{2}}+\frac{\Lambda }{2Ek_{1}^{3}}+\frac{2\Lambda }{Ek_{1}}+14\Lambda^{2}-\frac{\Lambda^{2}}{Ek_{1}^{2}}+\frac{9\Lambda^{3}}{2Ek_{1}}+\frac{9\Lambda^{4}}{2}=0. \end{array}$$

This polynomial cannot be solved in radicals, but a solution with elliptical functions is possible, e.g. by following the approach by Kiepert [91]. It is joyously presented in the appendix. In addition, Bairstow’s method [92] provides an approximation to the real-valued geostrophic and maximum branch as

3.21 $$\begin{array}{rl} k_{\text{geo2}} & \;=\frac{1}{36}(2^{1/3}Ek_{1}^{2/3}(-1808\Lambda^{4}+552\Lambda^{2}+9)-24Ek_{1}(576\Lambda^{5}-209\Lambda^{3}+3\Lambda )\\ & \;\;+18\times 2^{2/3}Ek_{1}^{-2/3}+66\times 2^{2/3}Ek_{1}^{1/3}(\Lambda -4\Lambda^{3})\\ & \;\;-36\times 2^{1/3}\Lambda \;Ek_{1}^{-1/3}-96\Lambda^{2}+18) \end{array}$$

and

3.22 $$\begin{array}{rl} k_{\text{max2}} & \;=4\Lambda^{2}+Ek_{1}(576\Lambda^{5}-209\Lambda^{3}+3\Lambda )\\ & \;\;+\sqrt{\frac{\Lambda }{Ek_{1}}}-1+\frac{\sqrt{Ek_{1}}(80\Lambda^{4}-24\Lambda^{2}-3)}{2\sqrt{\Lambda }}. \end{array}$$

As the initial guess for Bairstow’s method, we used the default quotients formed by coefficients of the highest powers of the polynomial (3.20), i.e. $a_{4}/a_{5}=1/2$ and $a_{3}/a_{5}=(2\Lambda /Ek_{1})+\Lambda^{2}+1$. Interestingly, during the first iteration, we obtain $k_{0}=\frac{1}{2}\left(\sqrt{4(2\Lambda /Ek_{1}+\Lambda^{2}+1)+\frac{1}{4}}+\frac{1}{2}\right)$, which captures the exact point where the geostrophic and the maximum branch intersect. We substitute $k_{o}$ into the quintic polynomial (3.20), allowing us to find the asymptotic upper bound of the magnetostrophic coexistence range (uc) and, thus, the Elbert range, viz.

3.23 $${\text{MG}}_{2}⇆{\text{MG}}_{3}:\;\Lambda_{\text{uc}}=\frac{1}{2}{\left(3^{4}\pi^{2}Ek\right)}^{-1/3}=\frac{1}{3}{\left(\frac{Ch}{2^{3}\pi^{2}}\right)}^{1/4}.$$

### (d) The magnetically dominated magnetostrophic range (${\mathrm{MG}}_{3}$)

The magnetostrophic mode $\left(a_{\text{ms}},Ra_{\text{ms}}\right)$ of equation (3.16) continues to exists for $\Lambda >\Lambda_{\text{uc}}$, whereas the geostrophic mode is no longer present. In this magnetically dominated magnetostrophic range (${\text{MG}}_{3}$), the Λ→∞ leading order solution is

3.24 $$a_{\text{ms2}}=\pi \Lambda^{1/2}\;\mathrm{and}\;Ra_{\text{ms2}}=\pi^{2}Ch.$$

The three magnetostrophic regimes elucidated above, ${\text{MG}}_{1}$, ${\text{MG}}_{2}$ and ${\text{MG}}_{3}$, only exist if $Ek<Ek^{*}$ (3.18).

### (e) The magnetic range (M)

If the Lorentz force dominates over the Coriolis force, i.e. formally in the limit of Λ→∞, a zeroth order series expansion of the polynomial (3.4) yields the known equation for magnetoconvection [9],

3.25 $$2k^{3}-3k^{2}-Ch_{1}=0.$$

The only real-valued, physical solution is

3.26 $$k_{\text{mag2}}=\frac{1}{2}\left(1+{\left(\sqrt{Ch_{1}}+\sqrt{Ch_{1}+1}\right)}^{-2/3}+{\left(\sqrt{Ch_{1}}+\sqrt{Ch_{1}+1}\right)}^{2/3}\right).$$

In the limit of Ch→∞, the leading order term of the critical wave and Rayleigh number are,

3.27 $$a_{\text{mag2}}=\frac{\pi^{2/3}}{2^{1/6}}Ch^{1/6}\;\mathrm{and}\;Ra_{\text{mag2}}=\pi^{2}Ch,$$

respectively. While the critical Rayleigh number is the same as in the ${\text{MG}}_{3}$ range, the wavenumber is different, this is also visible in figure 6. We define the border between these two ranges, $\Lambda_{m}$, by the condition $a_{\text{mag2}}=a_{\text{ms2}}$, resulting in

3.28 $${\text{MG}}_{3}⇆M:\;\Lambda_{m}=\frac{1}{2^{1/2}\pi }Ek^{-1/2}={\left(\frac{Ch}{2\pi^{2}}\right)}^{1/3}.$$

For fixed $Ek>Ek^{*}$ and varying Λ (figure 4), or for fixed $Ch<Ch_{\text{lc}}$ and varying Λ (figure 5), the geostrophic range (G) connects directly to the magnetic range (M), with no existing magnetostrophic regimes.

## 4. Linear stability of oscillatory rotating magnetoconvection

Our focus up till now has been on the Pr- and Pm-independent stationary onset modes of rotating magnetoconvection. However, for small Pr and Pm, convection can onset via time-dependent oscillatory motions, also known as overstability. The theory for oscillatory convection in rotating magnetoconvection is well developed [9,12,88]. We consider Pm≪Pr fluids, e.g. liquid metals, here. The resulting thermal–inertial oscillations are similar to the ones occurring in non-magnetic rotating convection [9,19,20,66,93,94]. Thus, we will summarize the results of the standard linear stability approach [9].

The critical wavenumber $a_{o}$, Rayleigh number $Ra_{o}$ and frequency $\omega_{o}$ for the oscillatory onset of rotating magnetoconvection are determined through minimization of the following equations [9,12]:

4.1 $$Ra=2\pi^{4}\frac{\pi^{2}+a^{2}}{a^{2}}\frac{{\left(\pi^{2}+a^{2}\right)}^{2}+\pi^{2}Ch}{{\left(\pi^{2}+a^{2}\right)}^{2}(1-Pr)-\pi^{2}\;Pr\;Ch}\left({\left(1+\frac{a^{2}}{\pi^{2}}\right)}^{2}+{\left(\frac{\omega Pr}{2\pi^{2}Ek}\right)}^{2}\right)$$

and

4.2 $$\omega^{2}=\frac{4\pi^{4}Ek}{\pi^{2}+a^{2}}\frac{{\left(\pi^{2}+a^{2}\right)}^{2}(1-Pr)-\pi^{2}Pr\;Ch}{{\left(\pi^{2}+a^{2}\right)}^{2}(1+Pr)+\pi^{2}Pr\;Ch}-4Ek^{2}\pi^{4}{\left(1+\frac{a^{2}}{\pi^{2}}+\frac{\pi^{2}Ch}{\pi^{2}+a^{2}}\right)}^{2}.$$

The oscillatory onset is then determined through $\partial_{k}Ra=0$, which leads to the nonic polynomial,

4.3 $$\begin{array}{rl} & k^{9}-\frac{3}{2}k^{8}+\frac{2Ch_{1}Pr}{Pr+1}k^{7}-\frac{Ch_{1}Ek_{1}^{2}(Pr+1)(8Pr+1)+Pr^{2}}{2Ek_{1}^{2}{\left(Pr+1\right)}^{2}}k^{6}-\frac{Ch_{1}^{2}Pr}{{\left(Pr+1\right)}^{2}}k^{5}\\ & \;\;-\frac{Ch_{1}Pr(Ch_{1}Ek_{1}^{2}(Pr+1)(6Pr+1)+Pr(2Pr+3))}{2Ek_{1}^{2}{\left(Pr+1\right)}^{3}}k^{4}\\ & \;\;+\frac{Ch_{1}Pr^{2}(1-2Ch_{1}^{2}Ek_{1}^{2}(Pr+1))}{Ek_{1}^{2}{\left(Pr+1\right)}^{3}}k^{3}\\ & \;\;+\frac{Ch_{1}^{2}Pr^{2}(Ch_{1}Ek_{1}^{2}-Pr)}{2Ek_{1}^{2}{\left(Pr+1\right)}^{3}}k^{2}-\frac{Ch_{1}^{4}Pr^{3}}{{\left(Pr+1\right)}^{3}}k-\frac{Ch_{1}^{4}Pr^{3}}{2{\left(Pr+1\right)}^{3}}=0. \end{array}$$

The polynomial (4.3) is reduced to ninth order, instead of the 12 order polynomial given by Eltayeb [12]. This lower-order polynomial results advantageously from our choice of $k=1+a^{2}/\pi^{2}$ instead of $a^{2}/\pi^{2}$.

The polynomial (4.3) is numerically straightforward to solve using current polynomial root finding algorithms. However, the original numerical calculations were time consuming and complicated. Indeed, these calculations were first made by Donna Elbert in the summer of 1955 during one of Chandrasekhar’s absences [53,88]. Further, Chandrasekhar made an error in the formulae, requiring Elbert to carry out these laborious calculations not once, but twice [53].

Unlike stationary rotating magnetoconvection, the oscillatory problem has a single, unique real-valued solution, $k_{o}$. The critical wavenumber is $a_{o}=\pi \sqrt{k_{o}-1}$ and the critical oscillatory frequency is calculated as

4.4 $$\omega_{o}^{2}=\frac{8k_{o}}{Ch_{1}Pr+k_{o}^{2}(Pr+1)}-\frac{4(Ek_{1}^{2}{\left(Ch_{1}+k_{o}^{2}\right)}^{2}+k_{o})}{k_{o}^{2}}.$$

Oscillatory convection is permitted only if $\omega_{o}^{2}>0$.

The critical Rayleigh number is given by

4.5 $$Ra_{o}=\frac{\pi^{4}k_{o}(Ch_{1}+k_{o}^{2})(4Ek_{1}^{2}k_{o}^{2}+\omega_{o}^{2}Pr^{2})}{2Ek_{1}^{2}(k_{o}-1)(Ch_{1}Pr+k_{o}^{2}(1-Pr))}.$$

We note that setting $\omega_{o}^{2}=0$ in (4.5) does not generally lead to the solution for stationary onset convection. The denominator in equation (4.5) shows that Pr needs to be less than unity for Ra to be positive, hence, 0<Pr<1. More specifically, requiring $\omega_{o}^{2}>0$, $Ch_{1}>0$ and $Ek_{1}>0$, equations (4.5) and (4.4) give the following restrictions

4.6 $$Ek_{1}<\frac{k^{1/2}}{Ch_{1}+k^{2}}\;\text{and}\;Pr<\frac{k^{2}(k-Ek_{1}^{2}{\left(Ch_{1}+k^{2}\right)}^{2})}{\left(Ch_{1}+k^{2})(k+Ek_{1}^{2}{\left(Ch_{1}+k^{2}\right)}^{2}\right)}<\frac{k^{2}}{Ch_{1}+k^{2}}.$$

**Figure 7.**
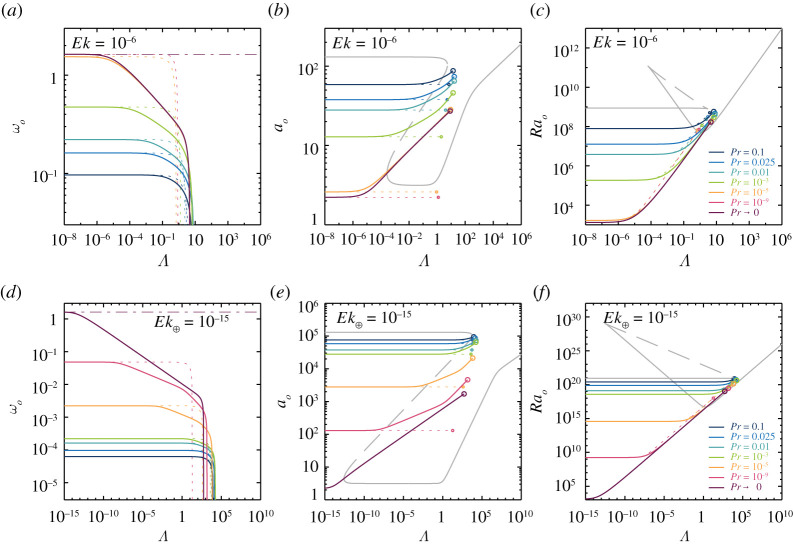
Onset predictions for oscillatory rotating magnetoconvection. The upper panels (*a*–*c*) show the predictions for $Ek=10^{-6}$, while the lower panels (*d*–*f*) show $Ek_{⊕}=10^{-15}$. The solid coloured line show the predictions for (*a*,*d*) the onset frequency $\omega_{o}$, (*b*,*e*) the critical wavenumber $a_{o}$, and (*c*,*f*) the critical Rayleigh number $Ra_{o}$ for Prandtl numbers $Pr\in \{10^{-9},10^{-5},10^{-3},0.01,0.025,0.1\}$ by solving equations (4.3)–(4.5). The dark purple line indicates the limit Pr→0 obtained by using the modified wavenumber (4.7). The dark purple dash-dotted lines in (*a*,*d*) indicate the upper bounding oscillation frequency of $\omega_{\text{up}}=\sqrt{\frac{8}{3}-9Ek^{2}}$. The dashed lines indicate the corresponding limit of Λ→0 using the modified wavenumber (4.9). For reference, the grey lines in (*b*,*c*,*e*,*f*) indicate the corresponding solutions for stationary rotating magnetoconvection. The open circles in (*b*,*c*,*e*,*f*) mark where $\omega_{o}=0$, as shown in (*a*,*d*); for higher Λ oscillatory linear solutions do not exist. (Online version in colour.)

The full numerical solutions of (4.3)–(4.5) for $\omega_{o}$, $a_{o}$ and $Ra_{o}$ depend on Pr, Ek and Ch. In particular, the smaller Ek, the lower the oscillation frequency, but the broader the Ch=Λ/Ek range in which oscillatory convection is possible, as demonstrated visually in figure 7. Note that the maximum permitted Pr for oscillatory rotating magnetoconvection differs from that of non-magnetic rotating convection, in which oscillatory solutions are found for Pr<0.6766 [88].

For Pr→0, we can express the asymptotic solution in terms of radicals. In the zeroth order series expansion in Pr, minimization of Ra only requires the solution of the cubic polynomial $Ch_{1}+(3-2k)k^{2}=0$. Since $Ch_{1}>0$, the discriminant $-108Ch_{1}(1+Ch_{1})$ is negative and, hence, the polynomial has one real and two non-real complex-conjugate roots. Only the real root is physically relevant and has critical wavenumber

4.7 $$k_{o}=\frac{1}{2}\left({\left(\sqrt{Ch_{1}}+\sqrt{Ch_{1}+1}\right)}^{-2/3}+{\left(\sqrt{Ch_{1}}+\sqrt{Ch_{1}+1}\right)}^{2/3}+1\right),$$

which can be used to calculate $\omega_{o}$, $a_{o}$ and $Ra_{o}$. We obtain an upper bound on the oscillation frequency when Ch→0, namely, $\omega_{\text{up}}=\sqrt{\frac{8}{3}-9Ek^{2}}$. For Pr→0 and using the restrictions in (4.6), oscillatory rotating magnetoconvection is only found to be possible for $\Lambda <{\left(2\pi^{2}Ek\right)}^{-1/5}$. However, for Pr>0, oscillatory convection is permitted for slightly larger Λ, and thus, it does not constitute an upper bound. Numerical evaluation further shows that the Pr→0 solutions do not accurately describe finite Pr solutions at small Ek, see figure 7*d*–*f*.

We can also consider the limit of Λ→0 since the oscillations originate from rotating and not magnetic overstability. This results in the following zeroth order $Ch_{1}$-independent cubic polynomial,

4.8 $$Ek_{1}^{2}{\left(Pr+1\right)}^{2}(2k-3)k^{2}-Pr^{2}=0.$$

The only real-valued solution is

4.9 $$\begin{array}{rl} k_{o} & \;=\frac{1}{2}(1+{(\frac{\xi }{\zeta^{2}})}^{1/3}+{(\frac{\zeta^{2}}{\xi })}^{1/3})\\ & \;\text{with }\zeta =Ek_{1}(1+Pr),\;\xi ={\left(Pr+\sqrt{Pr^{2}+\zeta^{2}}\right)}^{2}, \end{array}\}$$

and correspondingly calculated $\omega_{o}$, $a_{o}$ and $Ra_{o}$ are shown by the dashed lines in figure 7. As $Ek_{1}\to 0$, solution (4.9) approaches that of non-magnetic rotating convection [12,88], as is expected.

## 5. Linear stability of wall-attached rotating magnetoconvection

In confined containers, the onset of rotating magnetoconvection can also occur via wall-attached boundary modes, also known as wallmodes. These modes originate from the destabilizing effect of the sidewall and exponentially decay towards the interior. Thus, the bulk fluid remains virtually quiescent if no other instabilities are present [19,22]. Wallmodes have received considerable recent attention in the nonlinear regime of non-magnetic rotating convection [19–21,30,75,95–100]. They are, however, also of importance for rotating magnetoconvection [22]. The comprehensive linear theoretical framework for rotating magnetoconvective wallmodes was developed by Sánchez-Álvarez *et al.* [29] assuming a cylinder with zero curvature, i.e. a semi-infinite plane layer. Additional details are given in the supplementary Jupyter Notebook.

In the limit of Λ→0, the wallmodes behave as in rotating convection without magnetic field [29,66,86,101–104], showing Stewartson-layer characteristics, i.e. the critical frequency, wavenumber and Rayleigh number are given by

5.1 $$\begin{array}{rl} & \omega_{w}\to -{\left(2\pi \right)}^{2}{\left(6+3\sqrt{3}\right)}^{1/2}\frac{Ek}{Pr},\;a_{w}\to \pi {\left(2+\sqrt{3}\right)}^{1/2}\\ \mathrm{and}\; & Ra_{w}\to \pi^{2}{\left(6\sqrt{3}\right)}^{1/2}Ek^{-1}. \end{array}\}$$

Similarly, for Λ→∞, the wallmodes behave as in magnetoconvection without rotation [24,25,28,105], showing Shercliff-layer characteristics with the critical values

5.2 $$\omega_{w}\to 0,\;a_{w}\to \pi \sqrt{2}\;\mathrm{and}\;Ra_{w}\to 3\pi^{2}\sqrt{3\pi /2}Ch^{3/4}.$$

**Figure 8.**
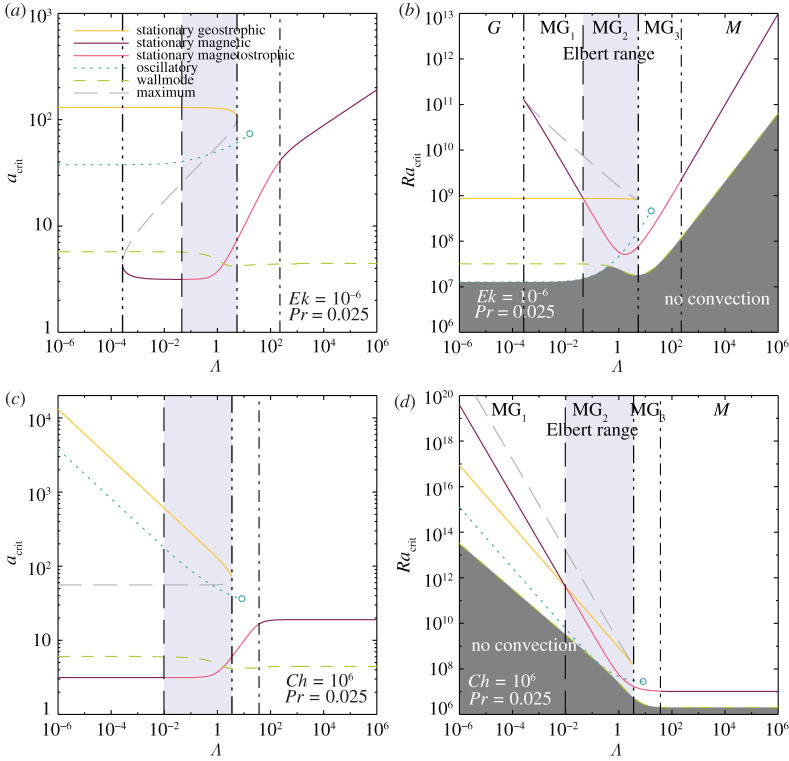
Laboratory predictions for multi-modal rotating magnetoconvection in a Pr=0.025 liquid metallic fluid. The Ekman number is held fixed at $Ek=10^{-6}$ in (*a*,*b*), whereas the Chandrasekhar number is held fixed at $Ch=10^{6}$ in (*c*,*d*). The semi-transparent cornflower blue region demarcates the Elbert coexistence range in each panel. The geostrophic range (G) does not exist in (*c*,*d*) because the fixed value of $Ch=10^{6}$ used there exceeds $Ch_{\text{lc}}=27\pi^{2}$ (3.11). The open blue circle in each panel marks where $\omega_{o}=0$; no oscillatory solutions exist at higher Λ. By contrast to the oscillatory and wallmodes, the stationary modes are independent of Pr. (Online version in colour.)

**Figure 9.**
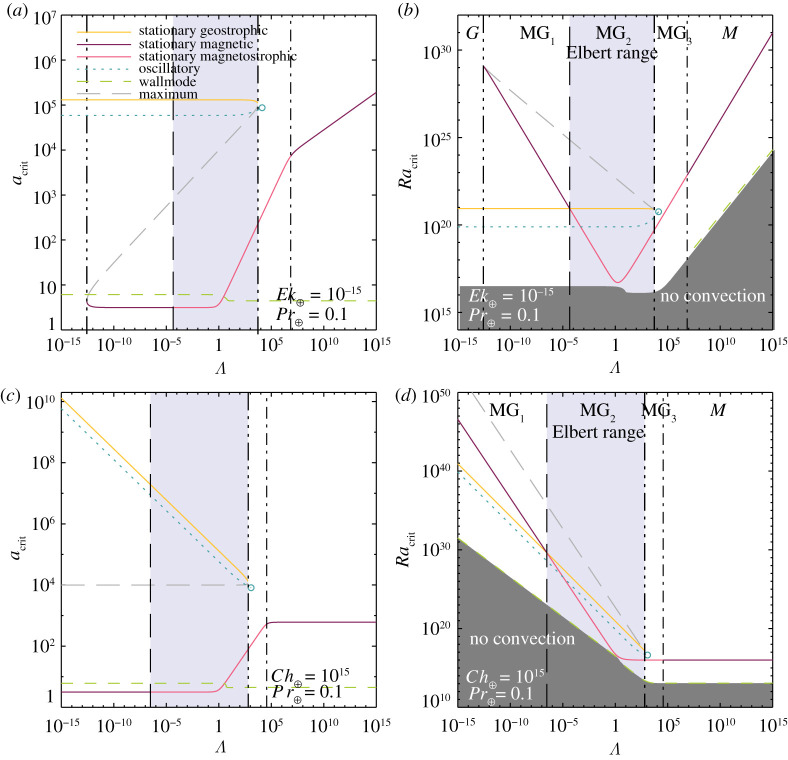
Earth’s core predictions for multi-modal rotating magnetoconvection in a $Pr_{⊕}=0.1$ core-like fluid. The Ekman number is held fixed at the Earth-like value $Ek_{⊕}=10^{-15}$ in (*a*,*b*), whereas the Chandrasekhar number is held fixed at the Earth-like value $Ch_{⊕}=10^{15}$ in (*c*,*d*). The semi-transparent cornflower blue region demarcates the Elbert coexistence range in each panel. The geostrophic range (G) does not exist in (*c*,*d*) because $Ch_{⊕}>Ch_{\text{lc}}$. The open blue circle in each panel marks where $\omega_{o}=0$; no oscillatory solutions exist at higher Λ. The stationary modes are independent of Pr, whereas the oscillatory and wall-attached modes are not. (Online version in colour.)

Importantly, because there is no horizontal magnetic symmetry breaking in this quasi-static framework, fully magnetic wallmodes do not drift, i.e. $\omega_{w}=0$. In the intermediate Λ≈1 magnetostrophic range, no asymptotic solution has been derived for rotating magnetoconvective wallmodes. It is only in this intermediate range that $\left(a_{w},Ra_{w}\right)$ vary as a function of Ek, Ch and Pr. Similarly to stationary rotating magnetoconvection, the critical Rayleigh number, $Ra_{w}$, attains a minimum value for magnetostrophic wallmodes [29]. However, the location of this minimum is shifted relative to $\Lambda_{\text{min}}=3^{1/2}$, equation (3.19). Typically the magnetostrophic wall mode well is broader, shallower and found at higher Λ values, as shown via the dashed green lines in figures 8*b* and 9*b*.

## 6. Summary and discussion

In this study, we have mapped out the regimes of liquid metal (Pm≪Pr) planar rotating magnetoconvection. There exists stationary, oscillatory and wall-attached linear convective modes. There are five distinct regimes of stationary onset: G, ${\text{MG}}_{1}$, ${\text{MG}}_{2}$, ${\text{MG}}_{3}$ and M (see table 1). The oscillatory branch only exists in the form of Coriolis-restored motions, since oscillatory modes cannot be driven by Lorentz forces in Pm→0 fluids. In finite geometries, the first instability occurs via wall-attached convection, since wallmodes have the lowest $Ra_{\text{crit}}$ in the vicinity of Λ∼1 (cf. [22]).

The stationary and oscillatory solutions are expressed in septic and nonic polynomial forms, (3.2) and (4.3), respectively. We solve them here using standard, fast and robust numerical root finding algorithms that did not exist in the 1950s when Chandrasekhar and Elbert first studied these systems. A Jupyter Notebook containing solutions that make use of this root finding approach is provided to the reader in the electronic supplementary material.

We have established an asymptotic framework for the five different regimes of stationary rotating magnetoconvection. Our framework yields novel analytical expressions that demarcate the boundaries between the regimes, i.e. (3.11) and (3.23). Further, when parsing the Λ-space, we have shown that all five regimes only exist when a fixed $Ek<Ek^{*}=16/{\left(27\pi \right)}^{2}$ is employed; for $Ek\geq Ek^{*}$, the geostrophic branch (G) connects directly to the magnetic branch (M) with no access to the multi-modal and magnetostrophic ${\text{MG}}_{1}$, ${\text{MG}}_{2}$ and ${\text{MG}}_{3}$ regimes (figure 4). By contrast, when parsing the Λ-space at a constant magnetic field strength Ch, two possible scenarios exist. The four regimes ${\text{MG}}_{1}$–${\text{MG}}_{3}$ and M exist if $Ch\geq Ch_{\text{lc}}=27\pi^{2}$. Otherwise, if $Ch<Ch_{\text{lc}}$, there are only the geostrophic (G) and the magnetic (M) branches (figure 5). Importantly, then, the Elbert range ${\text{MG}}_{2}$ does not exist for $Ek>Ek^{*}$, which leads us to argue that realistic models of planetary core convective processes (figure 1*b*) must be carried out at $Ek\ll Ek^{*}$ (e.g. [8,17,106], cf. [107–109]).

Figure 8 shows the entire zoo of functions characterized here under laboratory-like conditions. The Prandtl number, Pr=0.025, corresponds to that of a liquid metal [4]. In figure 8*a*,*b*, the angular rotation rate is held fixed such that $Ek=10^{-6}$ (e.g. [21,22]), whereas the imposed magnetic field strength is held fixed in figure 8*c*,*d* such that $Ch=10^{6}$ (e.g. [26,27]). The Elbert range ${\text{MG}}_{2}$ is shaded cornflower blue. The onset of the oscillatory, wall and stationary magnetostrophic modes all occur within an order of magnitude of each other, $10^{7}≲Ra_{\text{crit}}≲10^{8}$, in the vicinity of $\Lambda_{\text{min}}$. Further, the stationary geostrophic mode onsets at roughly one order of magnitude higher in Ra. Thus, ${\text{MG}}_{2}$ flows are expected to be richly multi-modal, possibly even more so than the multi-modality found in supercritical low Pr non-magnetic rotating convection (cf. [20]).

Figure 9 is similar to figure 8, but corresponds to Earth-like conditions, with Pr=0.1 [110,111] and the Ekman number held fixed at $Ek_{⊕}=10^{-15}$ in figure 9*a*,*b* and the Chandrasekhar number held fixed at $Ch_{⊕}=10^{15}$ in figure 9*c*,*d*. The Elbert range increases with decreasing Ek or increasing Ch, respectively, covering between eight and nine orders of magnitude in Λ under Earth-like conditions, whereas it covers roughly two orders of magnitude under laboratory conditions. The ordering of the critical Ra values differs near $\Lambda_{\text{min}}$ with the stationary magnetostrophic mode onsetting just after the wall mode instability. The oscillatory and geostrophic modes onset at higher supercriticalities.

It is this ordering of supercriticalities in figure 9 that has led to the mono-modal magnetostrophic dynamo hypothesis (e.g. [16], cf. [80,112]). However, estimates of the Rayleigh number for thermal convection in Earth’s core to lie roughly between $10^{23}≲Ra_{⊕}≲10^{29}$ [113,114] (figure 1*a*). Given this $Ra_{⊕}$ range, we expect that all of the possible modes in Elbert’s magnetostrophic coexistence range will be strongly excited in Earth’s core, similarly to what is achievable in turbulent laboratory experiments. This makes Elbert’s original discovery highly relevant for our understanding of planetary core rotating magnetoconvection, and likely for the dynamo action it generates. What remains to be answered is to what degree Elbert’s different modes are differentiable in remote indirect measures of core turbulence and which, if any, of them dominate over the others in strongly nonlinear, turbulent laboratory, planetary and astrophysical settings.

## Data Availability

The supplementary Jupyter Notebook allows the reader to recreate all our plots and, thus, access all the quantitative information presented in this manuscript.

## Appendix A. Solution of the Elbert range quintic polynomial

The quintic polynomial in the Elbert range, ${\text{MG}}_{2}$, is given by

A 1 $$\begin{array}{rl} p(x) & \;=x^{5}+\frac{1}{2}x^{4}+\left(1+\frac{2\Lambda }{Ek_{1}}+\Lambda^{2}\right)x^{3}+\left(2-\frac{1}{2Ek_{1}^{2}}+\frac{\Lambda }{2Ek_{1}}+\frac{5\Lambda^{2}}{2}\right)x^{2} \end{array}$$

A 2 $$\begin{array}{rl} & \;\;+\left(4-\frac{1}{Ek_{1}^{2}}+\frac{\Lambda }{Ek_{1}}+6\Lambda^{2}+\frac{\Lambda^{2}}{Ek_{1}^{2}}+\frac{2\Lambda^{3}}{Ek_{1}}+\Lambda^{4}\right)x \end{array}$$

A 3 $$\begin{array}{rl} & \;\;+8-\frac{2}{Ek_{1}^{2}}+\frac{\Lambda }{2Ek_{1}^{3}}+\frac{2\Lambda }{Ek_{1}}+14\Lambda^{2}-\frac{\Lambda^{2}}{Ek_{1}^{2}}+\frac{9\Lambda^{3}}{2Ek_{1}}+\frac{9\Lambda^{4}}{2}, \end{array}$$

where we replaced the wavenumber k by a general variable x. We further define

$$\begin{array}{rl} A & \;=\frac{1}{2}\\ B & \;=1+\frac{2\Lambda }{Ek_{1}}+\Lambda^{2}\\ C & \;=2-\frac{1}{2Ek_{1}^{2}}+\frac{\Lambda }{2Ek_{1}}+\frac{5\Lambda^{2}}{2}\\ D & \;=4-\frac{1}{Ek_{1}^{2}}+\frac{\Lambda }{Ek_{1}}+6\Lambda^{2}+\frac{\Lambda^{2}}{Ek_{1}^{2}}+\frac{2\Lambda^{3}}{Ek_{1}}+\Lambda^{4}\\ E & \;=8-\frac{2}{Ek_{1}^{2}}+\frac{\Lambda }{2Ek_{1}^{3}}+\frac{2\Lambda }{Ek_{1}}+14\Lambda^{2}-\frac{\Lambda^{2}}{Ek_{1}^{2}}+\frac{9\Lambda^{3}}{2Ek_{1}}+\frac{9\Lambda^{4}}{2}\\ \mathrm{and}\;p(x) & \;=x^{5}+Ax^{4}+Bx^{3}+Cx^{2}+Dx+E. \end{array}$$

We first apply a quadratic Tschirnhaus transformation

A 4 $$z=x^{2}+ux+v$$

to transform equation (A 2) to the principal quintic form

A 5 $$z^{5}+5lz^{2}+5mz+n=0.$$

The coefficient u in equation (A 4) is determined by any of the solutions to the quadratic equation

A 6 $$\begin{array}{rl} & \;(2A^{2}-5B)u^{2}+(4A^{3}-13AB+15C)u\\ & \;\;+(2A^{4}-8A^{2}B+10AC+3B^{2}-10D)=0, \end{array}\}$$

which yields

A 7 $$\begin{array}{rl} u & \;=-\frac{1}{2Ek_{1}(Ek_{1}(10\Lambda^{2}+9)+20\Lambda )}[-2Ek_{1}^{2}(31\Lambda^{2}+24)+11Ek_{1}\Lambda +15\\ & \;\;+\sqrt{5}\{Ek_{1}(Ek_{1}^{3}(-112\Lambda^{6}-28\Lambda^{4}+102\Lambda^{2}+45)-4Ek_{1}^{2}\Lambda (88\Lambda^{4}+443\Lambda^{2}+282)\\ & {\;\;-Ek_{1}(224\Lambda^{4}+183\Lambda^{2}+180)+64\Lambda^{3}+306\Lambda )+45\}}^{1/2}] \end{array}$$

and the coefficient v is determined through

A 8 $$5v=-Au-A^{2}+2B.$$

The coefficients l, m, n, are given by

A 9 $$\begin{array}{rl} 5l & \;=-C(u^{3}+Au^{2}+Bu+C)+D(4u^{2}+3Au+2B)-E(5u+2A)-10v^{3} \end{array}$$

A 10 $$\begin{array}{rl} 5m & \;=D(u^{4}+Au^{3}+Bu^{2}+Cu+D)-E(5u^{3}+4Au^{2}+3Bu2C)-5v^{4}-10lv \end{array}$$

A 11 $$\begin{array}{rl} \mathrm{and}\;n & \;=-E(u^{5}+Au^{4}+Bu^{3}+Cu^{2}+Du+E)-v^{5}-5lv^{2}-5mv. \end{array}$$

A second Tschirnhaus transformation

A 12 $$z=\frac{\alpha +\beta y}{y^{2}/\zeta -3}$$

then transforms the principal quintic to the Brioschi quintic [115] which only depends on the single parameter ζ,

A 13 $$y^{5}-10\zeta y^{3}+45\zeta^{2}y-\zeta^{2}=0.$$

(We could also depress the cubic term using an additional transformation to arrive at the Bring–Jerrard form.) The coefficient α is any of the roots of the quadratic equation

A 14 $$(l^{4}+lmn-m^{3})\alpha^{2}+(-11l^{3}m+ln^{2}-2m^{2}n)\alpha -27l^{3}n+64l^{2}m^{2}-mn^{2}=0,$$

where we chose

A 15 $$\begin{array}{rl} \alpha & \;=\frac{\left(11l^{3}m-ln^{2}+2m^{2}n\right)}{2(l^{4}+lmn-m^{3})} \end{array}$$

A 16 $$\begin{array}{rl} & \;+\frac{\sqrt{{\left(11l^{3}m-ln^{2}+2m^{2}n\right)}^{2}-4(l^{4}+lmn-m^{3})(-27l^{3}n+64l^{2}m^{2}-mn^{2})}}{2(l^{4}+lmn-m^{3})}, \end{array}$$

and the other coefficients in equation (A 12) are given by

A 17 $$\begin{array}{rl} \beta & \;=\frac{\gamma l^{2}-8\alpha^{3}l-72\alpha^{2}m-72\alpha n}{\alpha^{2}l+\alpha m+n}, \end{array}$$

A 18 $$\begin{array}{rl} \zeta & \;=\frac{1}{1728-\gamma }\;\text{with}\;\gamma =\frac{{\left(l\alpha^{2}-3m\alpha -3n\right)}^{3}}{l^{2}(ln\alpha -m^{2}\alpha -mn)}. \end{array}$$

The roots of the Brioschi quintic are given by

A 19 $$y_{r}=\frac{1}{\sqrt[{4}]{5}}{[(\xi_{\infty }-\xi_{r})(\xi_{r+2}-\xi_{r+3})(\xi_{r+4}-\xi_{r+1})]}^{1/2},\;r\in Z/5,$$

where $\xi_{\infty }$ and $\xi_{r}$ are the roots of the Jacobi sextic

A 20 $$\xi^{6}+\frac{10}{\Delta }\xi^{3}-\frac{12g_{2}}{\Delta^{2}}\xi +\frac{5}{\Delta^{2}}=0.$$

The roots of the Jacobi sextic can be obtained by evaluating the elliptic integral

A 21 $$\phi =\int_{℘(\phi )}^{\infty }\frac{\text{d}\psi }{\sqrt{4\psi^{3}-g_{2}\psi -g_{3}}}$$

which is the inverse to the Weierstraß elliptic function ℘(ϕ). The invariants $g_{2}$ and $g_{3}$ and the discriminant Δ are given by

A 22 $$g_{2}=\sqrt[{3}]{\frac{\left(1-1728\zeta \right)}{1728\zeta^{2}}},\;g_{3}=\sqrt{\frac{g_{2}^{3}-\Delta }{27}}\;\mathrm{and}\;\Delta =-\frac{1}{\zeta }.$$

The key quantity needed for solving the sextic and the quintic polynomial equation hereby is the corresponding half period ratio $\tau =\omega^{\prime }/\omega$, with the fundamental periods $\omega^{\prime }$ and ω of the elliptic integral (A 21). These periods are related to the Jacobi nome $h\equiv \exp(i\pi \tau )=\exp(i\pi \omega^{\prime }/\omega )$. Kiepert suggested to derive h through hypergeometric series as functions of the absolute invariant $g_{2}^{3}/\Delta$ [116,117]. However, here we follow a slightly different approach instead by calculating the half period ratio by means of the complete integral of the first kind K with the elliptic modulus k and the complementary elliptic modulus $k^{\prime }\equiv \sqrt{1-k^{2}}$,

A 23 $$\tau \equiv \frac{\omega^{\prime }}{\omega }=i\frac{K(k^{\prime })}{K(k)}=i\frac{K(\sqrt{1-k^{2}})}{K(k)}.$$

The elliptic modulus k can be expressed in terms of the roots of the cubic equation in the denominator of equation (A 21),

A 24 $$4\psi^{3}-g_{2}\psi -g_{3}=0.$$

The three roots are given by

A 25 $$\begin{array}{rl} e_{1} & \;=\frac{1}{2}\left(\frac{\sqrt[{3}]{\sqrt{3}\sqrt{27g_{3}^{2}-g_{2}^{3}}+9g_{3}}}{3^{2/3}}+\frac{g_{2}}{\sqrt[{3}]{3}\sqrt[{3}]{\sqrt{3}\sqrt{27g_{3}^{2}-g_{2}^{3}}+9g_{3}}}\right), \end{array}$$

A 26 $$\begin{array}{rl} e_{2} & \;=-\frac{\left(1-i\sqrt{3}\right)\sqrt[{3}]{\sqrt{3}\sqrt{27g_{3}^{2}-g_{2}^{3}}+9g_{3}}}{4\;3^{2/3}}-\frac{\left(1+i\sqrt{3}\right)g_{2}}{4\sqrt[{3}]{3}\sqrt[{3}]{\sqrt{3}\sqrt{27g_{3}^{2}-g_{2}^{3}}+9g_{3}}}, \end{array}$$

A 27 $$\begin{array}{rl} \mathrm{and}\;e_{3} & \;=-\frac{\left(1+i\sqrt{3}\right)\sqrt[{3}]{\sqrt{3}\sqrt{27g_{3}^{2}-g_{2}^{3}}+9g_{3}}}{4\;3^{2/3}}-\frac{\left(1-i\sqrt{3}\right)g_{2}}{4\sqrt[{3}]{3}\sqrt[{3}]{\sqrt{3}\sqrt{27g_{3}^{2}-g_{2}^{3}}+9g_{3}}}, \end{array}$$

and the modulus by

A 28 $$k=\sqrt{\frac{e_{2}-e_{3}}{e_{1}-e_{3}}}.$$

We then evaluate the complete elliptic integrals of the first kind K(k) and $K(k^{\prime })$ which can be easily done using e.g. mathematica with I EllipticK[1-k^2^]/EllipticK[k^2^]. Further using the Dedekind-η function defined as

A 29 $$\eta (\sigma )=\sum_{\lambda =-\infty }^{\infty }{\left(-1\right)}^{\lambda }\;e^{\sigma i\pi {\left(6\lambda +1\right)}^{2}/12},$$

and implemented as DedekindEta(σ) in Mathematica, the roots of the Jacobi sextic (A 20) read

A 30 $$\xi_{\infty }=\frac{5{\left[\eta (5\tau )\right]}^{2}}{\Delta^{1/3}{\left[\eta (h)\right]}^{2}}\;\mathrm{and}\;\xi_{r}=\frac{{\left[\eta ((1/5)(24r+\tau ))\right]}^{2}}{\Delta^{1/3}{\left[\eta (h)\right]}^{2}}.$$

In general it needs to be verified that the correct ordering of the roots $e_{1}$, $e_{2}$ and $e_{3}$ is used, as there are six possible permutations of {1,2,3}, and the correct roots are used, i.e. the following needs to hold:

A 31 $$(\xi -\xi_{\infty })\prod_{r=0}^{4}(\xi -\xi_{r})=\xi^{6}+\frac{10}{\Delta }\xi^{3}-\frac{12g_{2}}{\Delta^{2}}\xi +\frac{5}{\Delta^{2}}.$$

However, this is the case for k, $e_{1}$, $e_{2}$ and $e_{3}$ defined by equations (A 25)–(A 28) for our specific polynomial equation (A 3).

Now using equation (A 19)

A 32 $$\begin{array}{rl} y_{r} & \;=\frac{1}{\sqrt[{4}]{5}}\frac{1}{\Delta \eta^{6}(h)}[\left(5\eta^{2}(5\tau )-\eta^{2}(\frac{24r+\tau }{5})\right) \end{array}$$

A 33 $$\begin{array}{rl} & \;\;\times \left(\eta^{2}(\frac{24(r+2)+\tau }{5})-\eta^{2}(\frac{24(r+3)+\tau }{5})\right) \end{array}$$

A 34 $$\begin{array}{rl} & \;\;\times \left(\eta^{2}(\frac{24(r+4)+\tau }{5})-\eta^{2}(\frac{24(r+1)+\tau }{5})\right)] \end{array}$$

and transforming the roots according to equation (A 12),

A 35 $$z_{r}=\frac{\alpha +\beta y_{r}}{y_{r}^{2}/\zeta -3},$$

the general solution to the quintic, and thus, also to equation (A 3), is given by

A 36 $$x_{r}=-\frac{E+(z_{r}-v)(u^{3}+Au^{2}+Bu+C)+{\left(z_{r}-v\right)}^{2}(2u+A)}{u^{4}+Au^{3}+Bu^{2}+Cu+D+(z_{r}-v)(3u^{2}+2Au+B)+{\left(z_{r}-v\right)}^{2}}.$$
